# Angular Velocity, Moment, and Power Analysis of the Ankle, Knee, and Hip Joints in the Goalkeeper's Diving Save in Football

**DOI:** 10.3389/fspor.2020.00013

**Published:** 2020-02-28

**Authors:** Rony Ibrahim, Idsart Kingma, Vosse de Boode, Gert S. Faber, Jaap H. van Dieën

**Affiliations:** ^1^Sport Science Program, College of Arts and Sciences, Qatar University, Doha, Qatar; ^2^Department of Human Movement Sciences, Faculty of Behavioural and Movement Sciences, Vrije Universiteit Amsterdam, Amsterdam Movement Science, Amsterdam, Netherlands; ^3^Adidas miCoach Performance Centre, AFC Ajax, Amsterdam, Netherlands

**Keywords:** proximal-to-distal, football, biomechanics, strength and conditioning coach, sports performance

## Abstract

The aim of this study was to identify biomechanical characteristics of goalkeeper's diving performance in football. Lower extremity joints powers, moments, and angular velocities, were investigated in seven elite goalkeepers diving to save balls, shot from a ball canon to unanticipated heights (high and low) and sides (right and left). Our result showed that there was a proximal-to-distal sequence for each leg in timing of peak joints powers (*p* < 0.05). Hip extensors produced the largest (*p* < 0.05) peak moment, the contralateral (relative to dive side) peak was significantly larger than the ipsilateral one for high (4.56 ± 1.02 N·m·kg^−1^, and 3.52 ± 0.79 N·m·kg^−1^) and low dives (3.52 ± 0.79 N·m·kg^−1^, and 2.52 ± 0.56 N·m·kg^−1^). The ankle plantar flexors produced the second largest peak moment (*p* < 0.05), and the peak ipsilateral ankle power and angular velocity were the largest (*p* < 0.05) of all joints, during high (1,502 ± 338 W, and 14.73 ± 1.36 rad·s^−1^) and low dives (868 ± 263 W, and 14.14 ± 3.09 rad·s^−1^). Strength and conditioning coaches need to focus on hip extensors and ankle plantar flexors, and for specificity in power training that should elicit triple extension of the lower limbs' joints in a proximal-to-distal sequence.

## Introduction

Goalkeepers in football have the most specialized role in their team. Their actions require timed and explosive adjustments of body speed, position and orientation in response to a stimulus. One of their most critical tasks is defending the goal during a penalty shot, which is usually performed as a diving save. The diving save can be categorized as a defensive jumping skill, with a main objective to propel the body in the air through an explosive push-off, to reach and deflect ball trajectory. The push-off that is present in these skills, is a common pattern found in jumping movements. Previous biomechanical studies that observed jumping movements have described the push-off to be executed in a proximal-to-distal sequence (Bobbert and van Ingen Schenau, 1988; Pandy and Zajac, 1991; Chiu et al., 2014). As movement was found to start with the hip joint, then progressed to the knee and finally to the ankle joint. However, these studies looked at jumping tasks performed through simultaneous push-offs of both legs, which cannot be directly inferred and extended to the sequential push-offs seen in the goalkeeper's diving save (Suzuki et al., 1987; Spratford et al., 2009; Ibrahim et al., 2019a). The goalkeeper usually executes the diving save by first pushing-off with the contralateral leg, followed by the ipsilateral leg, which is different from the vertical jump, where both legs push-off simultaneously without any significant time delay between them (Ibrahim et al., 2019a).

Goalkeeper's coaching practices, whether from technical or strength and conditioning (S&C) coaches, are currently based on studies of vertical jumping and qualitative observations of the diving save rather than quantitative descriptive studies. Up to our knowledge, the study of Ibrahim et al. (2019a) was the first to address this gap, by conducting a full kinematic and kinetic analysis of the push-offs during the diving save and finding that the contribution of the contralateral push-off to the center of mass (COM) velocity was greater than the ipsilateral push-off. In addition, it was recommended that training horizontal sideward skills could be more specific to the diving save performance, as horizontal linear momentum was found to be larger than the vertical one. However, more details on the mechanics of the dive are still needed to develop guidelines for training goalkeepers. Lower body joints power, defined as joint moments times joint angular velocities, are considered by many to be important determinants of performance in sports that require the triple extension, which is extension of the hips, knees and ankles (Newton and Kramer, 1994; Zink et al., 2006; Hori et al., 2007, 2008). In addition, optimal training for the development of lower body power should adhere to the principle of specificity, which means that to maximize transfer, the exercises chosen should show similarities to the task itself in aspects such as musculature involved, movement pattern, movement velocity, and range of motion (Sheppard et al., 2016). Therefore, the aim of this study was to identify biomechanical characteristics of goalkeeper's performance during high and low unanticipated diving saves. Specifically, we compared timing and magnitude of moments, angular velocities and powers at the ankle, knee and hip joints between high and low and left and right dives. In addition, we strived in this study to improve the experimental set-up relative to previous studies by examining the diving save in a more realistic set-up, where the balls were shot from a custom-made ball canon instead of hanging them in a stationary position.

Based on the empirical findings of previous studies on the coordination pattern in vertical jumping task, we first hypothesized that goalkeepers generate joints power during the push-off of each leg in a proximal-to-distal sequence. Second, because a previous study found that the contralateral leg contributed more than the ipsilateral leg to the total COM velocity (Ibrahim et al., 2019a), we hypothesized that the total power of the contralateral leg to be larger than the ipsilateral leg. Third, because of the main frontal plane nature of the diving save, we hypothesized that hip abduction/adduction would be the largest contributor to the diving save performance, in terms of peak moment and power.

## Materials and Methods

Seven elite football goalkeepers, mean ± standard deviation age 18.9 ± 3 years, mass 84.9 ± 8.1 kg, height 186.5 ± 2.1 cm, and dominant leg determined as the shooting leg 6 right and 1 left, participated in this study. The participants' level, at the time of the experiment, was as follows: two goalkeepers competed in the Dutch Eredivisie (the highest level of competition nationally), three goalkeepers in the Dutch Eerste Divisie (the second highest level of competition nationally), and two goalkeepers in the Dutch under-17 Eredivisie (the highest level of competition nationally for players under 17 years of age). Before performing the experiment participants, or their parents, signed an informed consent form. For each participant, anthropometric measurements, age and injury history were gathered. Participants had not suffered from any injury that prevented them from performing the diving save at their maximum power or caused them to change their movement pattern at the time of the experiment. The experiments were conducted at the Adidas miCoach Performance Centre of AFC Ajax. The Ethics Committee of the Faculty of Behavioral and Movement Sciences of the Vrije Universiteit Amsterdam had approved the research protocol.

### Data Collection and Pre-processing

Before starting the measurement, the participants performed a goalkeeper specific warm-up routine with their coaches and around 8 diving saves to get familiar with the experimental set-up. Each participant was then instructed to react and dive as fast as possible to save the ball that was shot by a ball canon. For each participant, two successful dives were measured for two heights (high and low) at both sides of the goal, for a total of 8 successful dives per participant with 2 min recovery time between dives. A dive was considered successful when the goalkeeper dived and saved the ball by either hitting it or grabbing it. The order of the dives was randomized for each subject.

**Figure 1:**
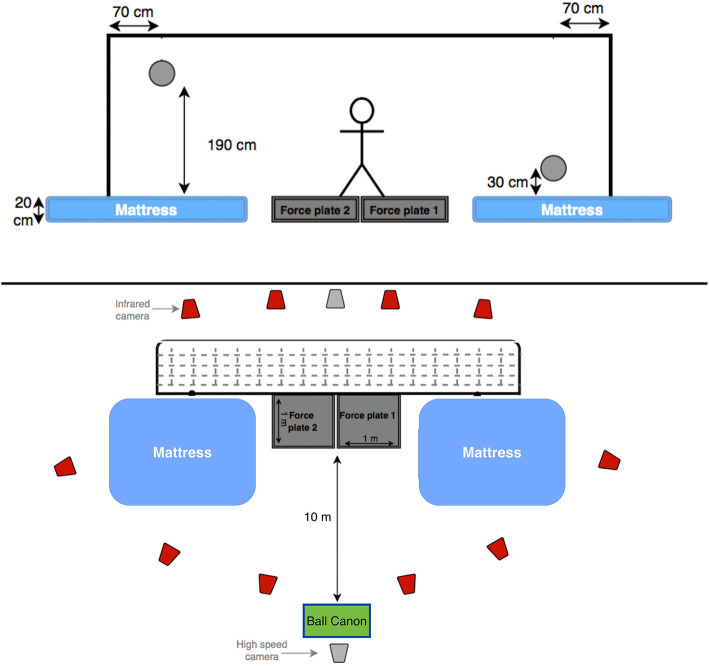
Two schematic diagrams of the experimental set-up (front and top view). The projected positions of the high and low balls are also presented.

The ball canon was placed at the penalty mark, and the front-end was covered with a very lightweight striped curtain, in order to prevent any anticipation of ball height and side. The ball canon was calibrated for the four goal corners (high and low corners at the right and left side of the goal) before every subject, and was not displaced during the whole measurement (Figure 1). The set ball speed was calculated to allow the ball to reach the goal in 1.2 ± 0.1 s, in accordance with the result of a recent study on total dive time (reaction time + dive movement time) by Ibrahim et al. (2019a). During the ball canon calibration, we aimed the ball to reach positions similar to a previous study by Ibrahim et al. (2019a), ~70 cm medially from the side post, and ~30 cm high for low balls and ~190 cm high for high balls from the force plates' level. The variability in the end-position of the ball (at ball contact) was found to be relatively small, with an average standard error of ±7 cm horizontally and ±6 cm vertically. Therefore, it was determined to be suitable and reliable for our analyses, which does not involve comparison of dive times.

A passive marker motion analysis system (Vicon 612, Oxford, UK), consisting of 10 infrared cameras, was used to capture, at 200 Hz, 3D coordinates data of 44 markers. Markers were attached to different body segments in the form of clusters (feet, shanks, pelvis, thorax, head, and forearms), the thighs were modeled between the shanks and pelvis, and the upper arms were modeled between the thorax and forearms, in order to obtain a full-body model without occlusion of the markers during the trials and limiting the risk of landing on markers. The markers were attached in a well-recognizable pattern to facilitate the labeling with Vicon Nexus Software (version 1.8.5). Soft markers were used on areas that are prone to impact at landing. Anatomical coordinate systems of the segments were marked with single markers and related to the corresponding marker clusters during a measurement in a reference position (T-pose). Details of the 3-D inverse dynamics model that was used in this study can be found elsewhere (Kingma et al., 1996; Faber et al., 2011, 2013; Ibrahim et al., 2016).

Two custom-made strain-gauge, 1 × 1 m, force plates (Vrije Universiteit Amsterdam, Amsterdam, The Netherlands) were used to measure ground reaction forces (GRF) produced by each leg separately at a rate of 1,000 Hz. Each force plate was separately covered by artificial football grass, to prevent any force transfer between force plates. A mattress was placed beside each force plate, to make the floor level even with the force plates, and to keep the goalkeeper and the marker set-up safe at each landing. Two Basler video cameras (50 Hz) were used to record all trials in the frontal plane for visual checks and for detection of ball contact.

### Data Analysis

All kinematic and kinetic analyses were carried out using custom software in MATLAB (R2015b, MathWorks Inc., US). A bi-directional second order low-pass Butterworth filter with a cut-off frequency of 12 Hz was used, to smooth the kinematic signals. The optimal cut-off frequency was estimated on kinematic data using the equation developed by Yu et al. (1999). Timing variables were defined relative to the onset of the dive, which was detected using an algorithm based on the Approximated Generalized Likelihood-Ratio (AGLR) (Staude and Wolf, 1999). AGLR was successfully used before for detecting the onset of the dive toward hanging balls (Ibrahim et al., 2019a,b). It works by (1) detecting the alarm time (the time instant when the signal reaches the pre-set threshold) using a sliding test window, then (2) tracking back the signal to detect the initial change time using Maximum Likelihood techniques (Poor, 1988). We used a threshold equal to 20% of the goalkeeper's body weight, and three different input signals [i.e., total horizontal GRF, total vertical GRF, and Vertical GRF of the contralateral leg (the leg opposite to the diving side)]. The dive onset was defined as the average of the two out of three onsets, having the smallest mutual difference.

The instants of contralateral (CPF) and ipsilateral peak force (IPF) were defined as the instants when the contralateral and ipsilateral leg exerted their maximum resultant GRF, respectively. Take-off was defined as the instant that the vertical component of GRF, summed over legs, dropped below 10% body weight and ball contact as the first frame when contact took place between the ball and the goalkeeper, as detected from the high-speed cameras.

The angular velocities of lower body joints (hips, knees and ankles) were calculated by first expressing the rotation matrix of the distal segment relative to the proximal one and then using the equation of Berme and Cappozzo (1990). Positions of the centers of mass and the moments of inertia were estimated according to Zatsiorsky (2002). Kinematics of the body segments were used together with the GRFs to calculate moments at the ankles, knees and hips, in a bottom-up dynamic linked segment model (Kingma et al., 1996). To obtain the 3D components of the net moments, the ankle moments were projected onto the foot coordinate system (CS), the knee moments were projected onto the shank CS, and the hip moments were projected onto the thigh CS. Hip, knee, and ankle powers were calculated by scalar multiplication of angular velocity and moment of the concerned joint. Thereafter, total power per leg was calculated by summing the power across the three joints for each leg.

Hip joint angles were defined as the Euler angles of the thigh to the pelvis anatomical coordinate systems. The sequence of rotation was: flexion-extension, external-internal rotation and abduction-adduction (Wu et al., 2002).

### Statistical Analysis

All time series were time-normalized (NT) from the detected movement onset to take-off. All data are presented as mean ± standard deviation. The timing and magnitude of peak joints power, and the magnitude of peak net joints moment, and of peak joints angular velocity were compared between joint movement planes, between dive heights (high and low) and sides (right and left) with three-way repeated measures ANOVAs. Joint movement plane was a factor of 8 levels: Contralateral hip flexion-extension, ipsilateral hip flexion-extension, contralateral hip adduction-abduction, ipsilateral hip adduction-abduction, contralateral knee flexion-extension, ipsilateral knee flexion-extension, contralateral ankle flexion-extension, ipsilateral ankle flexion-extension. If the results of three-way ANOVA showed a significant main effect for joint movement plane, pairwise comparisons were used to identify between which specific joint the timing of peak power differed significantly from the nearby peak in another joint.

The magnitude of peak power per leg was averaged over diving side and compared between legs (contralateral and ipsilateral), dive height (high and low) with two-way repeated measures ANOVA.

The level of significance was set at *p* < 0.05 and the effect size measure partial eta-squared was reported (0.01 small, 0.06 medium, 0.14 large). All statistical analyses were carried out using IBM SPSS Statistics 20.

## Results

The goalkeepers executed high and low dives using similar movement patterns in the push-off. They initiated the dive by side stepping with the ipsilateral leg toward the target, pushing-off with the contralateral leg and finally pushing-off with the ipsilateral leg.

**Figure 2:**
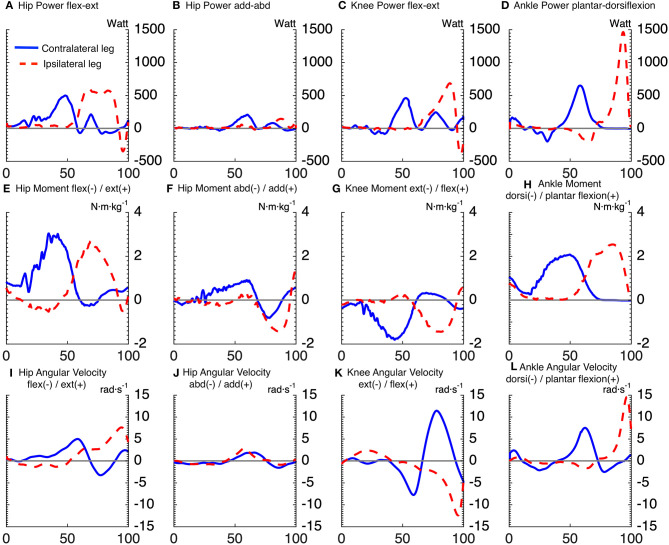
Time series averaged over subjects, for joints powers **(A–D)**, joints moments **(E–H)**, and joints angular velocities **(I–L)**, of goalkeepers diving to save high balls. Solid lines correspond to the contralateral leg, and the dashed lines to the ipsilateral leg. The x-axis of all subplots is the normalized time expressed in [%]. The titles **(A–D)** of joints powers indicate the plane of movement of the joint and not the direction of joint rotation. The sign of joints powers reflects power generation (+) and absorption (-).

**Figure 3:**
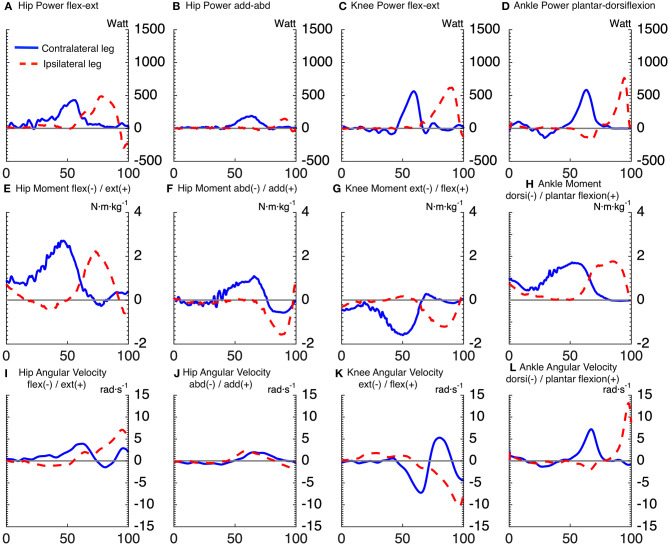
Time series averaged over subjects, for joints powers **(A–D)**, joints moments **(E–H)**, and joints angular velocities **(I–L)**, of goalkeepers diving to save low balls. Solid lines correspond to the contralateral leg, and the dashed lines to the ipsilateral leg. The x-axis of all subplots is the normalized time expressed in [%]. The titles **(A–D)** of joints powers indicate the plane of movement of the joint and not the direction of joint rotation. The sign of joints powers reflects power generation (+) and absorption (–).

Figures 2, 3 show the time series of lower body joint powers, moments and angular velocities from dive onset to take-off, during high and low dives, respectively. Contralateral push-off lasted from 16 to 75% NT during high dives, and from 23 to 83% NT during low dives. Whereas, the ipsilateral push-off lasted from 52 to 100% NT during high dives, and from 53 to 100% NT during low dives.

Repeated measures ANOVA for peak joint powers magnitude showed a main effect for joint movement plane (*p* < 0.001, large effect size = 0.78) and for dive height (*p* < 0.01, large effect size = 0.92), with no effect for dive side. Additionally, repeated measures ANOVA for peak joint moments magnitude showed a main effect for joint movement plane (*p* < 0.001, large effect size = 0.84) and for dive height (*p* < 0.05, large effect size = 0.79), with no effect for dive side. In addition, repeated measures ANOVA for peak joint angular velocities magnitude showed a main effect for joint movement plane (*p* < 0.001, large effect size = 0.95), with no effect for dive side and height. The contralateral hip flexion-extension power was induced by a large hip extension moment, which was the largest joint moment of all (*p* < 0.05). However, the ipsilateral hip flexion-extension power was characterized by a large area under the curve, from 63 to 86% of NT. This was induced by an ipsilateral hip extension moment, which was the second largest peak of all (*p* < 0.05). The peak ipsilateral ankle dorsi-plantar flexion power was significantly greater than all the other analyzed joint powers. It was induced by the fourth largest joint moment (ipsilateral ankle plantar-flexion moment), with the third largest being also plantar flexion but of the contralateral ankle (*p* < 0.05). As for the peak power per leg (ipsilateral vs. contralateral leg), there was a significant interaction between dive height and leg side (*p* < 0.05). During high dives, the peak power generated by the ipsilateral leg (2,294 ± 273 W) was significantly greater (*p* < 0.05) than the contralateral leg (1,846 ± 292 W). However, there was no significant difference between the peak powers generated by the ipsilateral (1,536 ± 291 W) and contralateral (1,643 ± 326 W) legs during low dives.

**Figure 4:**
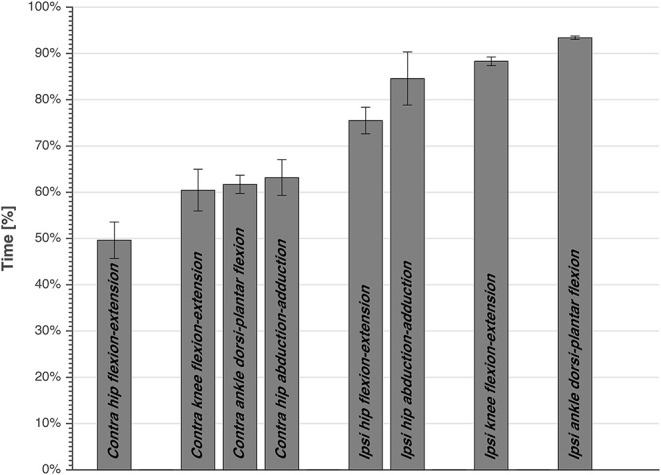
The timing of peak joints power averaged over subjects, side, and height, expressed as percentage of total time from dive-onset to take-off. Standard error is also presented. The bars are grouped, and each joint power of a group is significantly different than the other joints power of other groups.

Repeated measures ANOVA showed a significant effect of joint movement plane on timing of peak powers (*p* < 0.001, large effect size = 0.88), with no significant effect for dive side or height. Largely in line with the hypothesized proximo-distal sequence, pairwise comparisons showed that the sequence of lower limbs peak joints power consisted of 5 main events (Table 1, Figure 4): (1) peak contralateral hip flexion-extension power, (2) peak contralateral knee flexion-extension power, peak contralateral ankle dorsi-plantar flexion power, peak contralateral hip abduction-adduction power, (3) peak ipsilateral hip flexion-extension power, and abduction-adduction power, (4) peak ipsilateral knee flexion-extension power, and (5) peak ipsilateral ankle dorsi-plantar flexion power. The timing of each event number (1–5) was significantly different from the timing of the previous and the next event number.

**Table 1:** Timing and Magnitudes of peak joint power, along with the underlying magnitudes of peak net joint moment and angular velocity, and statistical results of three-way repeated measures ANOVA.

**Variables**		**Hip flexion-extension**		**Hip abduction-adduction**		**Knee flexion-extension**		**Ankle dorsi-plantar flexion**	
		**Contra-lateral**	**Ipsi-lateral**	**Contra-lateral**	**Ipsi-lateral**	**Contra-lateral**	**Ipsi-lateral**	**Contra-lateral**	**Ipsi-lateral**
High dives	Timing of peak joint power [% of NT]	46 ± 12	74 ± 9	62 ± 11	77 ± 26	59 ± 13	87 ± 3	60 ± 4	93 ± 0.5
	Peak joint power [W]	787 ± 220	860 ± 259	305 ± 122	360 ± 284	617 ± 231	727 ± 258	837 ± 174	1502 ± 338
	Peak net joint moment [N·m·kg^−1^]	4.56 ± 1.02	3.71 ± 0.62	2.11 ± 0.79	1.51 ± 0.38	2.22 ± 0.8	1.56 ± 0.49	2.75 ± 0.74	2.63 ± 0.31
	Peak joint angular velocity [rad·s^−1^]	5.62 ± 1.4	8.06 ± 1.38	3.14 ± 0.73	2.41 ± 1.47	9.48 ± 1.55	12.62 ± 1.31	9.74 ± 1.94	14.73 ± 1.36
Low dives	Timing of peak joint power [% of NT]	53 ± 9	77 ± 5	64 ± 9	93 ± 11	62 ± 10	89 ± 2	64 ± 6	94 ± 1
	Peak joint power [W]	656 ± 275	605 ± 181	318 ± 113	284 ± 210	543 ± 399	668 ± 229	658 ± 251	868 ± 263
	Peak net joint moment [N·m·kg^−1^]	3.52 ± 0.79	2.52 ± 0.56	1.81 ± 0.68	1.81 ± 0.29	1.76 ± 0.71	1.32 ± 0.28	1.98 ± 0.66	1.94 ± 0.24
	Peak joint angular velocity [rad·s^−1^]	5.18 ± 1.61	7.42 ± 1.61	3.23 ± 0.52	2.58 ± 2.3	9.37 ± 2.37	10.7 ± 1.87	8.38 ± 2.98	14.14 ± 3.09
Significant differences	Peak Power	High > Low		None		None		Ipsi > Contra High > Low	
	Peak net joint moment	Contra > Ipsi High > Low		None		Contra > Ipsi		High > Low	
	Peak joint angular velocity	Ipsi > Contra High > Low		None		Ipsi > Contra		Ipsi > Contra	
	Sequence (1–5)	(**1**)	(**3**.a)	(**2**.c)	(**3**.b)	(**2**.a)	(**4**)	(**2**.b)	(**5**)

*The bottom section of the table is showing the spotted significant differences for the tested factors (dive height, dive side, and leg side/joint movement plane). Sequence (1–5) groups the peak joint power by order of occurrence, in a way that each group is significantly different than the neighboring one(s). Subgroups (a, b, c) are assigned to peak joints powers that were not significantly different than each others (e.g., 3.b occurred non-significantly after 3.a)*.

For the first event, pairwise comparisons (Table 1) showed that peak hip joint power in the sagittal plane, hip extension moment and angular velocity were greater for high dives than low dives (*p* < 0.05). The contralateral hip generated also more extension moment than the ipsilateral hip (*p* < 0.05), whereas ipsilateral hip extension angular velocity was greater than the contralateral one (*p* < 0.05). During this first phase, we also calculated hip abduction-adduction and internal-external rotation angles at the moment of peak hip joint power, in order to look at the orientation of the goalkeeper. We found that these angles were almost zero, indicating a neutral posture in the frontal and transversal plane.

A result similar to the first event was also found for the second and fourth event, as the contralateral knee generated more extension moment (*p* < 0.05), and less extension angular velocity (*p* < 0.05) than the ipsilateral knee. Ipsilateral ankle power in the sagittal plane (fifth event) was significantly greater (*p* < 0.05) than the contralateral one (in the second event), and the same was evident for the resulting peak ankle plantar-flexion angular velocity (*p* < 0.05). Finally, during high dives peak ankle power in the sagittal plane and plantar-flexion moment were greater than during low dives (*p* < 0.05).

## Discussion

The current study was the first to attempt analyzing the biomechanics of goalkeeper's diving save in football, while simulating the penalty diving save as realistic as possible. In accordance with our first hypothesis, both the contralateral and ipsilateral legs followed roughly a proximal-to-distal sequence in peak joint powers. This is in agreement with findings of Chiu et al. (2014), analyzing lower limb coordination in a vertical jump task. They found that a proximal-to-distal sequence allows the athlete to generate larger hip extensor, knee extensor and ankle plantar flexor net joint moments, resulting in larger angular accelerations and pelvis linear acceleration. It was also suggested that, if net joint moments of hip and knee extensors occur concurrently, this may result in antagonist co-contraction at the knee, resulting in slower joint angular accelerations and slower pelvis linear acceleration. After analyzing the sequence in peak joints powers, we identified five main events in the diving save (Table 1, Figure 4): (1) peak contralateral hip flexion-extension power, (2) peak contralateral knee flexion-extension power, peak contralateral ankle dorsi-plantar flexion power, peak contralateral hip abduction-adduction power, (3) peak ipsilateral hip flexion-extension power, and abduction-adduction power, (4) peak ipsilateral knee flexion-extension power, and (5) peak ipsilateral ankle dorsi-plantar flexion power. The timing of the five identified events of peak joint power were significantly different from each other, however during the second event several joints reached their peaks sequentially, but without a significant difference in timing. In addition, the peak contralateral hip abduction-adduction power (2.c; Table 1) was unexpectedly the last peak for the contralateral leg, and the only joint rotation that did not respect the proximal-to-distal sequence. Contralateral hip abduction/adduction might be needed to transfer body weight from the contralateral leg to the ipsilateral one (side stepping), instead of contributing to the actual push-off, given also that its net joint moment was the lowest compared to other joints. Furthermore, the relatively large standard deviation in the peak hip abduction-adduction power, and other peak joints powers, might reflect inter-individual technical differences in elite level goalkeepers.

The analysis of peak power per leg showed that there was no significant difference between legs when diving to save low balls. In contrast, the peak ipsilateral power was greater than the contralateral one when diving to save high balls. This is in contradiction with our second hypothesis, which was based on the findings of Ibrahim et al. (2019a). However, total power per leg and joint power were somehow misleading variables to look at, given the fact that the diving save is characterized by sequential push-offs, i.e., first the contralateral and then the ipsilateral leg, which contrasts with simultaneous push-off such as in the vertical jump. The roles of the ipsilateral and contralateral legs can be understood better when considering the components of joint power separately, i.e., joint moments and angular velocities, in Table 1. The contralateral leg started the dive with a push-off initiated by a hip extension moment and followed by a knee extension moment, both peak joint moments were significantly greater than the ipsilateral ones during high and low dives (*p* < 0.05). In addition, contralateral hip adduction moment and ankle plantar-flexion moment were mostly non-significantly greater or in some cases equal, but never smaller than the ipsilateral ones (Table 1). Therefore, in line with the findings of Ibrahim et al. (2019a), the joints of the contralateral leg produced larger moments than the ipsilateral ones, especially for the hip joint extension moment that, in this study, was found to be the main contributor to the dive performance. Furthermore, the greater ipsilateral leg power was due to the larger joint angular velocities reached in the ipsilateral leg (Table 1), and especially in the distal joints (i.e., ipsilateral ankle). Based on the kinetic link principle, the linear velocity of the proximal end in the linked segment model (i.e., the pelvis segment in this study) can contribute positively and promote each subsequent joint angular velocity produced in that model (Putnam, 1993). The contralateral leg initiated the dive and started to push-off from a static position, so without initial pelvis linear velocity, which was not the case for the ipsilateral leg. The ipsilateral leg started to push-off after that the pelvis developed a positive linear velocity toward the target due to the contralateral push-off. This may have led to the production of greater joint angular velocities and joint powers in the ipsilateral leg without requiring high muscle activation and joint moments (Putnam, 1993).

The large net hip extension moment indicates the importance of training hip extensor muscles during a push-off movement pattern, in line with a previous study on standing broad and vertical jumps (Robertson and Fleming, 1987). While, the knee extension and hip abduction/adduction moments were the lowest, which suggests that they are not the main contributors to diving save performance. This contradicts our third hypothesis, which was based on the finding that the horizontal component of the push-off force was larger than the vertical component (Ibrahim et al., 2019a). However, in the current study we showed that this horizontal COM velocity was mainly produced by the hip extensors and ankle plantar-flexors, instead of hip add/abductors, which was possible because the body was laterally inclined toward the diving side.

In previous work on goalkeepers of lower performance levels, goalkeepers were found to dive toward high balls by first making two crossover steps toward the ball side (Graham-Smith et al., 1999). In contrast, we found in the current study that elite goalkeepers did not make crossover steps before pushing-off during high dives. They initiated the dive by side stepping with the ipsilateral leg toward the target, pushing-off with the contralateral leg and finally pushing-off with the ipsilateral leg. Similar to our previous study with hanging balls (Ibrahim et al., 2019a), we did not find a significant effect of dive side on any of the variables analyzed (Table 1). The latter is in contradiction with the result of another previous study, where balls were hanging from the high-post above the goal line (Spratford et al., 2009). One of the limitations of the current study was the use of mattresses on the landing area, on both sides of the goal. However, the mattresses were necessary to make the floor level even with the force plates, and they were used to keep the goalkeeper and the marker set-up safe at each landing. It was also believed that the presence of mattresses would not affect performance or the diving save pattern, instead it would allow the goalkeeper to dive comfortably without worrying about the landing and the marker set-up.

In a previous study with hanging balls instead of a ball canon, we suggested that S&C coaches, and technical coaches need to highlight horizontal lateral skills, to both sides of the body, with emphasis on the push-off with the contralateral leg (Ibrahim et al., 2019a). In part, this was based on the finding that, in the diving save, there is a strong contralateral leg contribution to total COM velocity, and a large horizontal linear momentum. In the current study, we have found that the main joints rotations, by analyzing joints moments and powers, are mainly the hip extensors and ankle plantar flexors. While this initially seems to contradict the above-mentioned focus on the lateral skills, it should be stressed that asymmetry of ipsi- and contralateral leg flexion-extension power can result in major lateral motions. The analysis of hip abduction/adduction angle, and hip external/internal rotation angle at the instant of maximum hip extension power, revealed that this near to oblique body orientation is not created by major joint movements in the frontal or transverse planes. Instead, it may be created by a total body angular momentum, from the contralateral push-off and ipsilateral sidestep.

Therefore, goalkeepers are advised to work closely with qualified strength coaches for power development, by relying on the kinetic results of the current study combined with the kinematic results and recommendations of our previous study (Ibrahim et al., 2019a). Power exercises initiated by hip extension and followed by extension of the knee and ankle joints in a proximal-to-distal manner (e.g., power clean and hang power clean, power snatch and hang power snatch, and push jerk) need to be included in goalkeeper performance training (Baumann et al., 1988; Garhammer, 1993; Gourgoulis et al., 2000; Chiu and Schilling, 2005; Tricoli et al., 2005; Kipp et al., 2011). In addition, lateral movement patterns in the frontal plane driven by the hip extension of the contralateral leg (e.g., side push-offs, side sled pull, asymmetrical side squat), adhere to the principle of specificity and might insure maximum transfer from gym training to actual field performance Sheppard et al., 2016. Future intervention studies can test the effect of the above training recommendations along with the ones from our previous study (Ibrahim et al., 2019a), on goalkeeper's dive time.

In conclusion, goalkeepers perform the diving save using a proximal-to-distal sequence in lower extremity peak joint powers. Hip extension movement, especially the contralateral hip extension, generated the largest peak net joint moment during the dive. Overall, net joint moments in the contralateral leg reached larger peaks than the ipsilateral leg, in almost all main joint movements. However, joint angular velocities of the ipsilateral leg were larger than the contralateral ones, leading to larger peak total power for the ipsilateral leg during high dives and to similar peaks during low dives. These findings can be used to improve prescriptions of technical and strength training for goalkeepers.

## Data Availability Statement

The datasets analyzed in this article are not publicly available because the football clubs involved didn't give permission for data sharing. Requests to access the datasets should be directed to Rony Ibrahim, rony.r.ibrahim@gmail.com.

## Ethics Statement

The studies involving human participants were reviewed and approved by the Ethics Committee of the Faculty of Behavioral and Movement Sciences of the Vrije Universiteit Amsterdam. Written informed consent to participate in this study was provided by the participants' legal guardian/next of kin.

## Author Contributions

RI contributed to the whole study (literature review, research design, experimental set-up, measurements, data processing, data analysis, and writing the manuscript). IK contributed to the research design, experimental set-up, data analysis, and writing the manuscript. VB contributed to the research design, experimental set-up, and data processing. GF contributed to the research design and experimental set-up. JD contributed to the data analysis and writing the manuscript.

### Conflict of Interest

VB was employed by the company AFC Ajax. The remaining authors declare that the research was conducted in the absence of any commercial or financial relationships that could be construed as a potential conflict of interest.

## References

[B1] Baumann W. Gross V. Quade K. Galbierz P. Schwirtz A. (1988). The snatch technique of world class weightlifters at the 1985 world championships. Int. J. Sport Biomech. 4, 68–69. 10.1123/ijsb.4.1.68

[B2] Berme N. Cappozzo A. (1990). Biomechanics of Human Movement: Applications in Rehabilitations, Sports and Ergonomics. Worthington, OH: Bertec Corp, 89–97.

[B3] Bobbert M. F. van Ingen Schenau G. J. (1988). Coordination in vertical jumping. J. Biomech. 10, 249–262. 10.1016/0021-9290(88)90175-3 3379084

[B4] Chiu L. Z. Bryanton M. A. Moolyk A. N. (2014). Proximal-to-distal sequencing in vertical jumping with and without arm swing. J Strength Cond. Res. 28, 1195–1202. 10.1519/JSC.0000000000000388 24476777

[B5] Chiu L. Z. Schilling B. (2005). A primer on weightlifting: from sport to sports training. Strength Cond. J. 27, 42–48. 10.1519/00126548-200502000-00008

[B6] Faber G. S. Chang C. Kingma I. Dennerlein J. (2013). Lifting style and participant's sex do not affect optimal inertial sensor location for ambulatory assessment of trunk inclination. J. Biomech. 46, 1027–1030. 10.1016/j.jbiomech.2012.12.007 23394716

[B7] Faber G. S. Kingma I. van Dieën J. H. (2011). Effect of initial horizontal object position on peak L5/S1 moments in manual lifting is dependent on task type and familiarity with alternative lifting strategies. Ergonomics 54, 72–81. 10.1080/00140139.2010.535019 21181590

[B8] Garhammer J. (1993). A review of power output studies of Olympic and Power lifting: methodology, performance prediction, and evaluation tests. J. Strength Cond. Res. 7, 76–89. 10.1519/00124278-199305000-00002

[B9] Gourgoulis V. Aggeloussis N. Mavromatis G. Garas A. (2000). Three-dimensional kinematic analysis of elite Greek weightlifters. J. Sports Sci. 18, 643–652. 10.1080/02640410050082332 10972413

[B10] Graham-Smith P. Lees A. Richardson D. (1999). Analysis of technique of goalkeepers during the penalty kick. J. Sports Sci. 17, 905–929. 10610406

[B11] Hori N. Newton R. U. Andrews W. A. Kawamori N. McGuigan M. R. Nosaka K. (2007). Comparison of four different methods to measure power output during the hang power clean and the weighted jump squat. J. Strength Cond. Res. 21, 314–320. 10.1519/R-22896.1 17530989

[B12] Hori N. Newton R. U. Andrews W. A. Kawamori N. McGuigan M. R. Nosaka K. (2008). Does performance of hang power clean differentiate performance of jumping, sprinting, and changing of direction? J. Strength Cond. Res. 22, 412–418. 10.1519/JSC.0b013e318166052b 18550955

[B13] Ibrahim R. Faber G. S. Kingma I. van Dieën J. H. (2016). Kinematic analysis of the drag flick in field hockey. Sports Biomech. 16, 45–57. 10.1080/14763141.2016.1182207 27192924

[B14] Ibrahim R. Kingma I. de Boode V. Faber G. S. van Dieën J. H. (2019a). Kinematic and kinetic analysis of the goalkeeper's diving save in football. J. Sports Sci. 37, 313–321. 10.1080/02640414.2018.1499413 30036138

[B15] Ibrahim R. Kingma I. de Boode V. Faber G. S. van Dieën J. H. (2019b). The effect of preparatory posture on goalkeeper's diving save in football. Front Sports Active Living 1:15 10.3389/fspor.2019.00015 PMC7739625 33344939

[B16] Kingma I. de Looze P. M. Toussaint M. H. Klijnsma G. H. Bruijnen T. B. M. (1996). Validation of a full body 3-D dynamic linked segment model. Hum. Mov. Sci. 15, 833–860. 10.1016/S0167-9457(96)00034-6

[B17] Kipp K. Harris C. Sabick M. (2011). Lower extremity biomechanics during weightlifting exercise vary across joint and load. J. Strength Cond. Res. 25, 1229–1234. 10.1519/JSC.0b013e3181da780b 21240030

[B18] Newton R. Kramer W. (1994). Developing explosive muscular power: implications for a mixed methods training strategy. Strength Cond. J. 16, 20–31. 10.1519/1073-6840(1994)016<0020:DEMPIF>2.3.CO;2

[B19] Pandy M. G. Zajac F. E. (1991). Optimal muscular coordination strategies for jumping. J. Biomech. 24, 1–10. 10.1016/0021-9290(91)90321-d 2026629

[B20] Poor H. V. (1988). An Introduction to Signal Detection and Estimation. New York, NY; Berlin; Heidelberg: Springer, 173–185. 10.1007/978-1-4757-3863-6

[B21] Putnam C. A. (1993). Sequential motions of body segments in striking and throwing skills: descriptions and explanations. J. Biomech. 26, 125–135. 10.1016/0021-9290(93)90084-r 8505347

[B22] Robertson D. G. Fleming D. (1987). Kinetics of standing broad and vertical jumping. Can. J. Appl. Sport Sci. 12, 19–23. 3594313

[B23] Sheppard J. M. Triplett T. N. National Strength and Conditioning Association (U.S.) (2016). Program design for resistance training, in Essentials of Strength and Conditioning, 4th Edn. ed Triplett T. N. (Champaign, IL: Human Kinetics), 440.

[B24] Spratford W. Mellifont R. Burkett B. (2009). The influence of dive direction on the movement characteristics for elite football goalkeepers. Sports Biomech. 8, 235–245. 10.1080/14763140903229526 19891201

[B25] Staude G. Wolf W. (1999). Objective motor response onset detection in surface myoelectric signals. Med. Eng. Phys. 21, 449–467. 10.1016/s1350-4533(99)00067-3 10624741

[B26] Suzuki S. Togari H. Isokawa M. Ohashi J. Ohgushi T. (1987). Analysis of the goalkeeper's diving motion, in Proceedings of the First World Congress of Science and Football, ed Reilly T. (Liverpool: E&F Spon). 468–475.

[B27] Tricoli V. Lamas L. Carnevale R. Ugrinowitsch C. (2005). Short-term effects on lower-body functional power development: weightlifting vs. vertical jump training programs. J. Strength Cond. Res. 19, 433–437. 10.1519/R-14083.1 15903387

[B28] Wu G. Siegler S. Allard P. Kirtley C. Leardini A. Rosenbaum D.. (2002). ISB recommendation on definitions of joint coordinate system of various joints for the reporting of human joint motion, Part I: Ankle, hip and spine. J. Biomech. 35, 543–548. 10.1016/s0021-9290(01)00222-6 11934426

[B29] Yu B. Gabriel D. Noble L. An K. (1999). Estimate of the optimum cutoff frequency for the butterworth low-pass digital filter. J. Appl. Biomech. 15, 318–329. 10.1123/jab.15.3.318

[B30] Zatsiorsky V. M. (2002). Kinetics of Human Motion. Champaign, IL: Human Kinetics.

[B31] Zink A. J. Perry A. C. Robertson B. L. Roach K. E. Signorile J. F. (2006). Peak power, ground reaction forces, and velocity during the squat exercise performed at different loads. J. Strength Cond. Res. 20, 658–664. 10.1519/R-16264. 16937981

